# Description of a novel method for detection of sleep‐disordered breathing in brachycephalic dogs

**DOI:** 10.1111/jvim.16783

**Published:** 2023-05-26

**Authors:** Iida Niinikoski, Sari‐Leena Himanen, Mirja Tenhunen, Liisa Lilja‐Maula, Minna M. Rajamäki

**Affiliations:** ^1^ Department of Equine and Small Animal Medicine University of Helsinki Helsinki Finland; ^2^ Faculty of Medicine and Health Technology Tampere University Tampere Finland; ^3^ Department of Clinical Neurophysiology Tampere University Hospital, Medical Imaging Centre and Hospital Pharmacy, Pirkanmaa Hospital District Tampere Finland; ^4^ Department of Medical Physics Tampere University Hospital, Medical Imaging Centre and Hospital Pharmacy, Pirkanmaa Hospital District Tampere Finland

**Keywords:** apnea‐hypopnea index, at‐home device, brachycephalic obstructive airway syndrome, Obstructive Respiratory Event Index, obstructive sleep apnea

## Abstract

**Background:**

Sleep‐disordered breathing (SDB), defined as any difficulty in breathing during sleep, occurs in brachycephalic dogs. Diagnostic methods for SDB in dogs require extensive equipment and laboratory assessment.

**Objectives:**

To evaluate the usability of a portable neckband system for detection of SDB in dogs. We hypothesized that the neckband is a feasible method for evaluation of SDB and that brachycephaly predisposes to SDB.

**Animals:**

Twenty‐four prospectively recruited client‐owned dogs: 12 brachycephalic dogs and 12 control dogs of mesocephalic or dolicocephalic breeds.

**Methods:**

Prospective observational cross‐sectional study with convenience sampling. Recording was done over 1 night at each dog's home. The primary outcome measure was the obstructive Respiratory Event Index (OREI), which summarized the rate of obstructive SDB events per hour. Additionally, usability, duration of recording, and snore percentage were documented.

**Results:**

Brachycephalic dogs had a significantly higher OREI value (Hodges‐Lehmann estimator for median difference = 3.5, 95% confidence interval [CI] 2.2‐6.8; *P* < .001) and snore percentage (Hodges‐Lehmann estimator = 34.2, 95% CI 13.6‐60.8; *P* < .001) than controls. A strong positive correlation between OREI and snore percentage was detected in all dogs (*r*s = .79, *P* < .001). The neckband system was easy to use.

**Conclusions and Clinical Importance:**

Brachycephaly is associated with SDB. The neckband system is a feasible way of characterizing SDB in dogs.

**Abbreviations**
AHI/REI: Apnea Hypopnea Index/Respiratory Event Index
BCS: body condition score
BD: brachycephalic dog
BOAS: brachycephalic obstructive airway syndrome
CI: confidence interval
OREI: Obstructive Respiratory Event Index
OSA: obstructive sleep apnea
REM: rapid eye movement
SBD: sleep‐disordered breathing
WBBP: whole‐body barometric plethysmography

## 1 INTRODUCTION

Sleep‐disordered breathing (SDB) implies any abnormal breathing during sleep, ranging from hypopnea, that is, shallow breathing due to partial obstruction, to apnea, a complete cessation of airflow.^1^ In humans, SDB can be classified into obstructive sleep apnea (OSA), central sleep apnea, upper airway resistance syndrome, and sleep hypoventilation syndrome.^2^ In dogs, SDB closely resembling human OSA, characterized in sleep measurements by recurring episodes of hypopnea and apnea caused by upper airway obstruction, is described in English Bulldogs^3^, ^4^ and Cavalier King Charles Spaniels^5^ with signs of brachycephalic obstructive airway syndrome (BOAS). In brachycephalic dogs (BDs), the congenital reduction of the cranio‐facial length in the absence of a concurrent reduction in the soft tissues can lead to obstruction of upper airways that is, BOAS, and cause breathing difficulties also during sleep.^6^, ^7^, ^8^, ^9^, ^10^ Consequences of SDB reported in BDs include sleeping in a sitting position or with the chin elevated, snoring, perceived apneic episodes during sleep, and not being able to sleep.^8^, ^11^, ^12^, ^13^ In dogs with a higher body condition score (BCS), the risk for clinical signs of BOAS is higher.^14^ OSA results in fragmented sleep, sympathetic activation, and hypoxemia in people.^15^ The relationships between OSA and decreased quality of life, cardiovascular disease, systemic inflammation, and hypertension are extensively studied in humans.^16^, ^17^, ^18^, ^19^ Inflammatory changes resembling those seen in human OSA occur in BDs but not in connection with evaluation of SDB.^20^, ^21^, ^22^ Additionally, cardiovascular abnormalities bearing resemblance to those seen in OSA occur in BDs.^23^, ^24^, ^25^, ^26^

Although the diagnostics, comorbidities, consequences, and treatment of OSA in humans are well researched, knowledge of SDB in dogs is scarce. In people, the gold standard for diagnosis of SDB is polysomnography, a comprehensive sleep study performed in a sleep laboratory.^1^ However, when OSA is suspected, portable devices can be used for readily accessible and inexpensive at‐home screening.^27^, ^28^ In dogs, diagnostic methods are limited to polysomnography^3^ and whole‐body barometric plethysmography (WBBP).^5^ Both of these methods require extensive laboratory equipment and the dog must be able to sleep in a clinical setting. As untreated, severe OSA considerably increases the mortality and morbidity in humans,^29^ practical tools to enhance our understanding of SDB in dogs are needed. Easily available, convenient measurement devices offer opportunities for evaluating the prevalence, severity, consequences, and treatment options, including surgery and medical treatment, of SDB also in dogs.^5^, ^30^, ^31^

The objective of this study was to evaluate the usability of a novel portable at‐home device for the detection of SDB in dogs in their home environment. Our hypotheses were 2‐fold. First, that the device, a wearable neckband system validated for OSA screening in humans,^28^ would be a feasible and well‐tolerated method for detection of SDB in dogs. Second, that brachycephaly predisposes to SDB.

## 2 MATERIALS AND METHODS

### 2.1 Study subjects and protocol

The study protocol was approved by the Committee of Experimental Animals of Southern Finland (ESAVI/10906/04.10.07/2017, ESAVI/34278/15.11.21/2021) and by the University of Helsinki Viikki Campus Research Ethics Committee (13/2020, 11/2021).

This prospective, observational cross‐sectional study with convenience sampling was performed at the Veterinary Teaching Hospital, University of Helsinki, Finland and at the Kaarina Veterinary Clinic, Kaarina, Finland, between October 2020 and September 2021. All animals were privately owned pet dogs. The owners signed an informed consent form before participation.

Privately owned dogs were recruited to participate in the study. The inclusion criteria for all dogs were facial conformation, that is, brachycephalic and mesaticephalic or dolicocephalic, age over 1 year, and, to ensure neckband fit, weight over 9 kg. Detailed history was taken and pregnant dogs and dogs with medications affecting breathing during sleep, such as ondansetron and tricyclic antidepressants, or gastroesophageal reflux requiring treatment, were excluded. Dogs with illnesses other than those resulting from conformational changes in the upper respiratory tract or with medications not directly affecting breathing during sleep were not excluded.

A physical examination was performed and blood samples for health verification obtained during a study visit. A 9‐point scale was used for scoring BCS.

Owners were advised to place the portable neckband system developed for human OSA diagnostics (Nukute Ltd, Oulu, Finland) on their dog's neck at home for 1 night. The Nukute neckband system includes a c‐shaped neckband device, a pulse oximeter (Berry BM2000D, Shanghai Berry Electronic Technology Co Ltd, Shanghai, China), and a tablet that provides instructions and routes the data. The neckband combines a piezoelectric microphone for tracheal sounds, an ambient microphone, and a gyroscope providing information on movement and position. The device yields results closely matching data from conventional polysomnography in humans.^28^ The neckband is available in 4 sizes, suitable for neck girths between 25 and 65 cm. The audio was recorded with 16 000 Hz sampling rate and saved as 2‐channel 16‐bit integers. Gyroscope data was saved with 10 Hz sampling rate as 32‐bit integers. Breathing‐related signals were derived from the tracheal sound recordings and respiratory rate signal using audio. A picture of the neckband is presented in Data S1, Supporting Information.

The size of the neckband used was determined by the dog's neck girth. The pulse oximeter was not used during the recording, as the finger probe used with the device is not suitable for dogs. The owner was advised to place the neckband on the dog late in the evening and to remove it upon waking in the morning. Recorded time was defined as duration of recording. The minimum duration of recording was set at 2 hours to ensure sufficient rapid eye movement (REM) sleep. The owner kept notes of potential distractions during the night, and usability was evaluated by oral feedback.

Data were transferred to the manufacturer's analysis software and analyzed by an experienced sleep researcher (SLH). The apnea and hypopnea events were scored manually as in human children.^32^ Children's rules were chosen as dogs have a similarly high respiratory rate. Tracheal sounds were used to calculate airflow signal automatically, and respiratory events were scored on this computer derived airflow trace. Physiologic central apneas related to sighs and movements were not included in the analyses. The respiratory event results were summarized as the Respiratory Event Index (REI) value, which describes the number of apnea and hypopnea events per monitoring time. Central apneas were few and since the aim was to evaluate obstructive breathing, obstructive REI value (OREI) was used in further statistical analyses. The presence of snoring was based on the level of breathing noise, and an individual detection threshold was determined by listening to the audio signal of each dog. The percentage of snoring (as time spent snoring during recorded time) was manually scored and ensured by listening. A visual example of the data trace is presented in Data S2, Supporting Information.

### 2.2 Statistical analysis

Continuous data were assessed for normality using the Shapiro‐Wilk test. Normally distributed data are presented as mean ± SD and nonparametric data as median with range. Differences in nonparametric variables (OREI values and snore percentage) between BDs and control dogs, and between dogs aged under and over 5 years were analyzed with the Mann‐Whitney *U* test. The difference in parametric variable (duration of recording) between BDs and control dogs was analyzed with Student's *t*‐test. The correlation between OREI value and snore percentage was analyzed with the Spearman rank correlation.

All statistical analyses were done using GraphPad Prism for Macintosh, version 9.3.0 (GraphPad Software, San Diego, California). *P*‐values <.05 were considered statistically significant.

## 3 RESULTS

### 3.1 Demographics

The study group consisted of 12 BDs and 12 normocephalic (mesaticephalic or dolicocephalic) control dogs of different breeds. The BD group comprised 9 French Bulldogs and 1 of each of: English Bulldog, Cavalier King Charles Spaniel, and Bullmastiff. The control group comprised 3 Labrador Retrievers, 2 Golden Retrievers, 2 Basset Fauve de Bretagne, and 1 of each of: Lapponian Herder, Whippet, Wales Terrier, Spanish Waterdog, and Irish Setter. The demographics are presented in Table 1. The Bullmastiff and 1 French Bulldog had had surgical treatment for BOAS. Of the 24 dogs, 21 attended a study visit and had no notable changes, discounting BOAS‐related issues, on clinical examination, hematology, or serum biochemistry.

**TABLE 1:** Demographics of dogs participating in the study.

	Brachycephalic dogs (n = 12)	Control dogs (n = 12)
Sex		
Female	5	5
Neutered/intact	1/4	4/1
Male	7	7
Neutered/intact	1/6	2/5
Age (y)		
Median/range	4.4/2.0‐9.0	5.4/1.5‐10.1
Weight (kg)		
Median/range	12.7/9.8‐57.0	20.0/10.4‐37.8
Body condition score (1‐9)		
Median/range	4/4‐6	5/4‐6

### 3.2 Device usability

Usability feedback indicated that the device was easy to use and well tolerated by all dogs. All owners reported that the neckband system did not disrupt the dogs' sleep. Small‐scale technical issues with network connection were seen in 1 BD and 1 control dog, resulting in briefer recording durations.

### 3.3 Sleep‐disordered breathing variables

**FIGURE 1:**
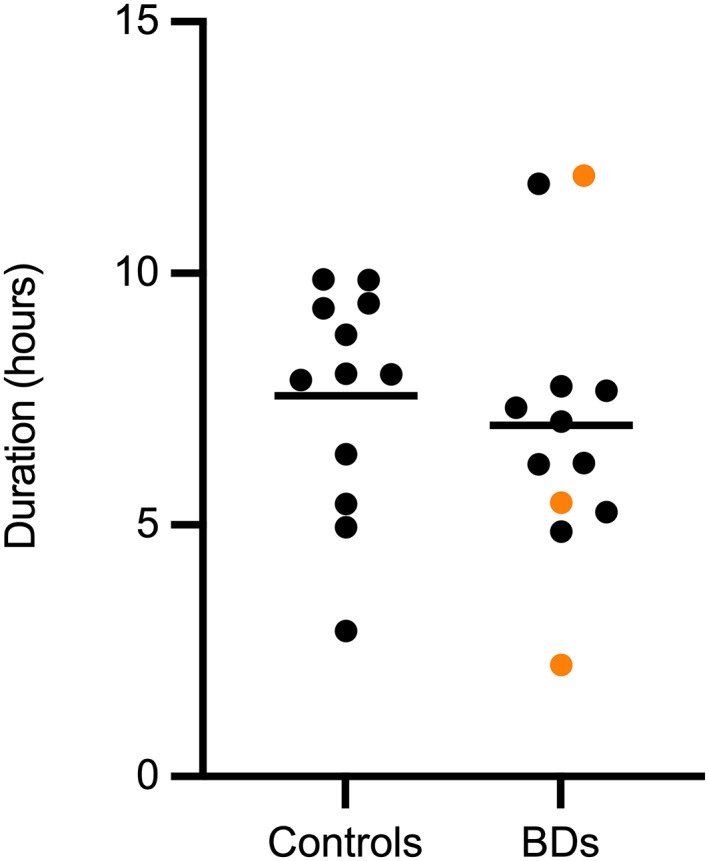
Scatter plot with mean duration of recording in hours in control dogs and brachycephalic dogs (BDs). No significant difference was found between the 2 groups. In BDs, the black data points denote French Bulldogs, and the orange data points denote other brachycephalic breeds.

The mean duration of recording was 6.98 hours (range, 2.22‐11.95; SD = 2.74) in BDs and 7.57 hours (2.90‐9.88; SD = 2.21) in controls. There was no significant difference in recording time between the groups (estimate for difference = 0.59, 95% CI −2.69 to 1.52, *P* = .57; Figure 1).

**FIGURE 2:**
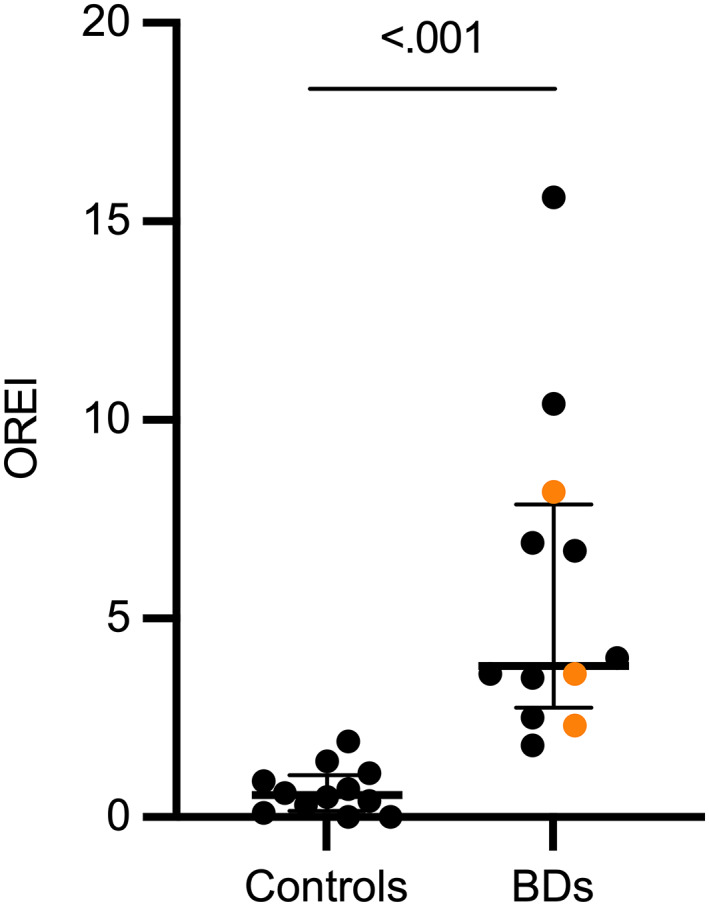
Scatter plot with median and interquartile range of obstructive Respiratory Event Index (OREI) in control dogs and brachycephalic dogs (BDs). In BDs, the black data points denote French Bulldogs, and the orange data points denote other brachycephalic breeds.

The BDs had a significantly higher OREI value than controls (median, 3.80; range, 1.80‐15.60 vs 0.55; 0.00‐1.90; Hodges‐Lehmann estimator for median difference = 3.5, 95% CI 2.2‐6.8; Figure 2).

**FIGURE 3:**
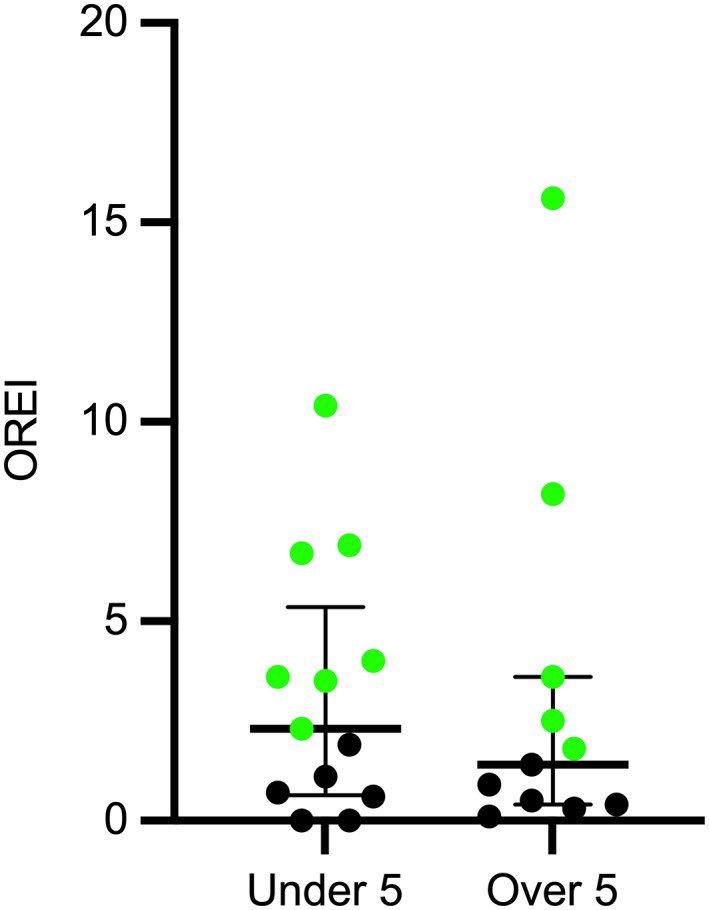
Scatter plot with median and interquartile range of obstructive Respiratory Event Index (OREI) in all 24 dogs aged under and over 5 years. No significant difference was found between the 2 groups. Green data points denote brachycephalic dogs.

No difference in OREI value was detected between dogs aged under 5 years and dogs aged over 5 years (median, 2.3; range, 0.0‐10.4 vs 1.4; 0.1‐15.6; Hodges‐Lehmann estimator for median difference = −0.4, 95% CI 3.1‐1.4; *P* = .70; Figure 3).

**FIGURE 4:**
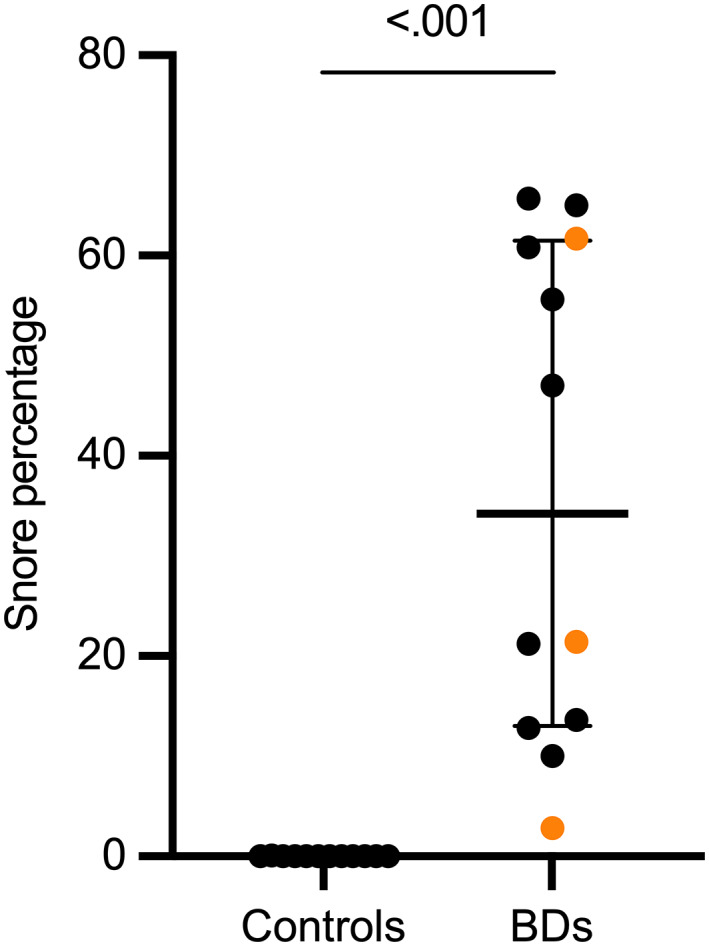
Scatter plot with median and interquartile range of snore percentage, as time spent snoring of total recording time, in control dogs and brachycephalic dogs (BDs). In BDs, the black data points denote French Bulldogs, and the orange data points denote other brachycephalic breeds.

Snore percentage was significantly higher in BDs than in controls (median, 34.2; range, 2.80‐65.70 vs 0.00; 0.00‐0.10; Hodges‐Lehmann estimator = 34.2, 95% CI 13.6‐60.8; Figure 4). A strong positive correlation between OREI value and snore percentage was detected in all dogs (*r*s = .79, *P* < .001), but not in BDs (*r*s = .15, *P* = .65).

## 4 DISCUSSION

We found the Nukute neckband system to be a well‐tolerated and feasible method of diagnosing SDB and snoring in dogs. BDs had higher OREI values, confirming earlier findings of brachycephaly predisposing to SDB. Snoring is common in BDs.

Due to the convenience sampling method, French Bulldogs were overrepresented in the BD group. The BD breed distribution, with extremely short skull conformation in French Bulldogs and the English Bulldog, might affect the results. The results would potentially be different if the BD group consisted of dogs with longer snouts such as the Bullmastiff and the Cavalier King Charles Spaniel. However, in addition to the laryngeal area, also obstruction of the nasal cavity can result in SDB, as documented in the Cavalier King Charles Spaniel.^5^ In questionnaire studies, English Bulldogs, French Bulldogs, Pugs, and Chihuahuas are distinguishable with signs of sleep disturbances.^8^, ^11^, ^12^, ^30^, ^31^, ^33^ The comorbid conditions or concurrent medications existing in our study group are not suspected of generating SDB.

Variation in BCS was low in both groups, and thus, its effect on the recorded variables was minimal. However, as there were no overweight or obese dogs (BCS ≥7) in either group, the effect of obesity could not be ascertained here. In humans, obesity is a well‐established risk factor for OSA, as prevalence of OSA increases with degree of obesity,^34^ and weight loss is an effective treatment form.^35^ In dogs, obesity has a negative effect on lung function of healthy dogs^36^ and is a risk factor for BOAS.^9^ In people, neck fat accumulation and large neck circumference are strongly associated with OSA.^37^, ^38^ Although the relationship between neck girth ratio and upper airway obstruction in BDs is unknown, greater neck girth increases the risk of BOAS.^14^, ^39^

The Nukute neckband system offers a feasible tool for detection of SDB in dogs. The device was well tolerated by dogs and easy to use for owners and could be a useful diagnostic method for SDB in dogs. Portable at‐home devices have obvious advantages compared with expensive and cumbersome polysomnography, which is the gold standard for SDB diagnostics. Previously, polysomnography, including measurement of neural activity, eye movements, muscle activity, heart rhythm, and respiratory function, was used for SDB measurement in a small cohort of English Bulldogs,^3^, ^4^ and later in sleep macrostructure and cognitive studies in normocephalic dogs.^40^, ^41^, ^42^ Whole‐body barometric plethysmography, the other method earlier applied successfully for SDB detection, has been used in 3 Cavalier King Charles Spaniels.^5^ In WBBP, the dog rests in the WBBP chamber and barometric pressure oscillations proportional to tidal volume induced by respiration are analyzed. Measurement is affected by panting, which prevented analysis in 2 of 5 dogs.^5^ Both of these methods require extensive equipment, and the dog must be able to sleep in a laboratory environment. Polysomnography is nevertheless the only alternative that yields information on sleep stages and sleep quality in dogs; the Nukute neckband system does not provide these data.

The duration of recording did not differ between the 2 groups. The advised recording time for people is overnight, but in our study technical issues with network connectivity shortened the recording in 2 cases. In contrast to generally monophasic human sleep, where sleep occurs in 1 long period, sleep in dogs is polyphasic and occurs in various smaller periods with time between periods spent awake.^43^, ^44^ The sleep stages in dogs are drowsiness, non‐REM sleep, and REM sleep,^40^ and the sleep cycle during which all sleep stages are experienced is about 20 minutes.^44^ The obstruction occurs mainly at REM sleep,^4^ and thus, acceptable minimum recording time in our study was set at 2 hours to allow for at least 1 complete polyphasic sleep‐wake period, including a REM sleep phase.^43^, ^44^, ^45^

The OREI value was significantly higher in BDs than in controls, indicating SDB. Sleep disturbances observed by owners are reported in BDs.^8^, ^11^, ^12^, ^13^ Apnea and hypopnea events can be caused by either obstructive or central conditions. Obstructive apneas are thought to result from the obstruction of upper airways due to loss of muscle tone during REM sleep phase.^3^, ^4^ Additionally, myopathic changes, including morphologically abnormal muscle fibers and increased connective tissue, are reported in upper airway dilator muscles of English Bulldogs, further impairing their activity during sleep.^46^

The range of OREI values in BDs was wide, from 1.8 to 15.6, which are considered low to moderate values in humans.^1^ These OREI values probably reflect the group of BDs not presenting for surgical treatment of BOAS, that is, the less severe cases. Noteworthy is that normocephalic dogs had minimal variation, with OREI values between 0 and 1.9. Previously, a SDB index closely resembling the OREI value presented here has been calculated from polysomnography recordings in English Bulldogs.^3^ The English Bulldogs in the studies by Hendricks et al^3^, ^4^ were more seriously affected by SDB than our dogs, and the SDB index values ranged from 0.5 to 114, with markedly more SDB events in REM sleep. As in our study, the control dogs had almost no SDB events, with values ranging from 0 to 0.9.^3^ However, the OREI and SDB index values are not entirely comparable, as values measured by polysomnography are considerably more exact since wake periods can be excluded from the analysis. In polysomnography in humans, the Apnea Hypopnea Index/Respiratory Event Index (AHI/REI) value is used to summarize the number of apneas and hypopneas per hour of sleep.

The diagnostic criteria for OSA in humans include AHI/REI value greater than 15 on its own or greater than 5 combined with unintentional sleep episodes during wakefulness; daytime sleepiness; unrefreshing sleep; fatigue; insomnia; waking up breath holding, gasping, or choking; or loud snoring or breathing interruptions, or both.^1^ In stark contrast to adults, obstructive AHI/REI over 1 is abnormal in children.^47^ As the OREI value has not been used in large‐scale studies in dogs, defining thresholds between normal and abnormal as well as severity grading of SDB in dogs warrant further investigations. However, in our study group, an OREI of 2 seemed to distinguish between brachycephalic and normocephalic dogs.

Both obstructive and central apneas are described in English Bulldogs, but neither their proportion nor count are reported.^3^ In humans, central apneas can occur in combination with obstructive apneas or independently as a result of, for instance, neurological disease or heart failure.^48^, ^49^, ^50^ Central apneas occur when the brain's respiratory control center fails to send signals needed for respiration, and no respiratory effort is made. As central apneas cannot be detected using WBBP and in the study by Hinchliffe et al^5^ all 5 Cavalier King Charles Spaniels responded positively to corrective upper airway surgery, it was concluded that obstruction of the laryngeal region and nose due to aberrant turbinates contributed at least partly to the SDB events. Although the control dogs represented a plethora of normocephalic breeds of various sizes, the OREI values and also snore percentages are convergent. It seems that conformational changes in the upper airways, not breed or size, affect SDB in dogs.

We found no significant difference in OREI values between dogs aged under 5 years and dogs aged over 5 years across both groups. However, the oldest brachycephalic dog in the study was only 9 years old, and therefore, age‐related changes in SDB remain unknown. In humans, the prevalence of OSA increases with age,^51^, ^52^, ^53^ with later onset for women.^52^ As BOAS can progress with age, secondary changes, such as eversion of laryngeal saccules, laryngeal collapse, and tonsillar hypertrophy, could further worsen respiratory obstruction^54^ and aggravate SDB.

All BDs, but none of the normocephalic dogs, snored. Snoring results from soft tissue vibration during sleep, and it is often caused by changes in the upper airway properties leading to obstruction of the upper airways.^55^ In BDs, an increased degree of pharyngeal narrowing is associated with the severity of snoring, while soft palate length alone is not.^56^ Similar findings are described in earlier studies, where all English Bulldogs^3^, ^4^ and Cavalier King Charles Spaniels^5^ snored, while the control dogs did not.^3^, ^4^ However, the proportion of time spent snoring during sleep has not earlier been objectively measured. Previously published owner‐reported prevalence of sleep disturbances, including snoring, in BDs varies between 2.7% and 66.0%.^8^, ^11^, ^12^, ^13^ In questionnaire studies, however, the presence of snoring or other sleep disturbances can be difficult to detect anamnestically, as owners can consider snoring normal for the brachycephalic breeds and not regard it as a sign of conformational changes associated with brachycephaly.^33^

We found snoring to correlate positively with OREI values in all dogs but not within BDs, suggesting that the amount of time spent snoring is not indicative of the degree of SDB in BDs. Additional diagnostics, such as use of the Nukute neckband system, are thus needed to exclude SDB in BDs.

This study has some limitations. Due to the pre‐defined neckband sizes available, only dogs with a suitable neck girth were recruited to the study. The device cannot currently be used in small or toy breeds with a neck girth of less than 25 cm. A broader sample size is needed to investigate differences between dogs with varying degrees of BOAS severity. No obese dogs participated in our study, and thus, the effect of obesity on SDB could not be evaluated.

SDB events in dogs can be detected by using a portable neckband system in the dog's home surroundings. Brachycephaly predisposes to SDB. The neckband system is well accepted by dogs and easy for owners to use for dogs with a neck girth suitable for the device.

## CONFLICT OF INTEREST DECLARATION

SLH acts as medical advisor for Nukute Ltd. The authors have no conflicts of interest to declare; none of the authors has a financial relationship with Nukute Ltd.

## OFF‐LABEL ANTIMICROBIAL DECLARATION

Authors declare no off‐label use of antimicrobials.

## INSTITUTIONAL ANIMAL CARE AND USE COMMITTEE (IACUC) OR OTHER APPROVAL DECLARATION

The study protocol was approved by the Committee of Experimental Animals of Southern Finland (ESAVI/10906/04.10.07/2017, ESAVI/34278/15.11.21/2021) and by the University of Helsinki Viikki Campus Research Ethics Committee (13/2020, 11/2021).

## HUMAN ETHICS APPROVAL DECLARATION

Authors declare human ethics approval was not needed for this study.

## Supporting information


**Supporting information S1.** A picture of the neckband device.

**Supporting information S2.** Data trace from the neckband system.
