# Supplementary figures and images for: Description of a novel method for detection of sleep‐disordered breathing in brachycephalic dogs
Source: J Vet Intern Med. 2023 May 26;37(4):1475–81. doi: 10.1111/jvim.16783 (PMC10365046; doi:10.1111/jvim.16783)

Supporting information S1. A picture of the neckband device.

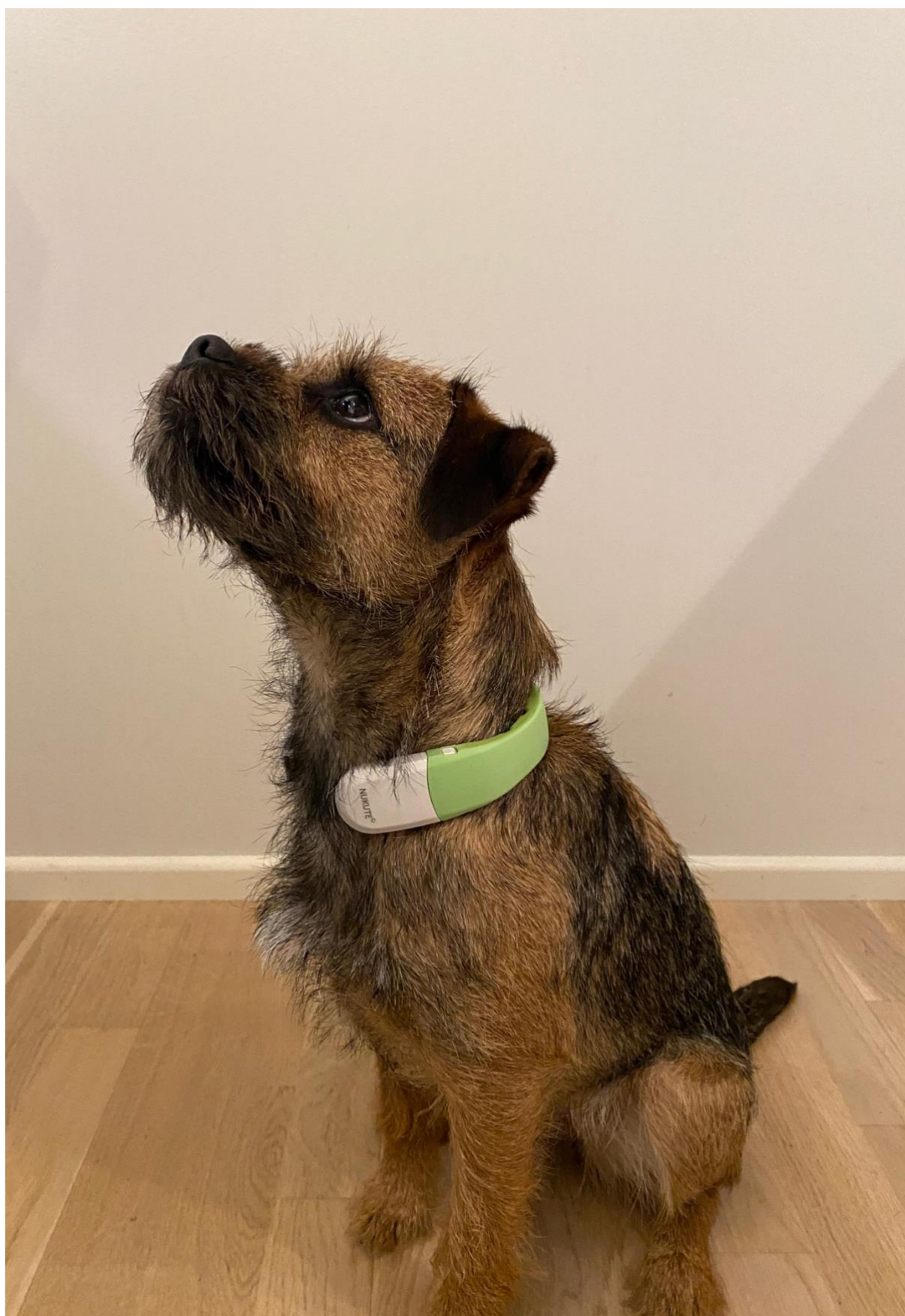

Supplement: Supplementary file 1 — Supporting information S1. A picture of the neckband device. [file JVIM-37-1475-s001.pdf]
