# Supplementary material for: Description of a novel method for detection of sleep‐disordered breathing in brachycephalic dogs
Source: J Vet Intern Med. 2023 May 26;37(4):1475–81. doi: 10.1111/jvim.16783 (PMC10365046; doi:10.1111/jvim.16783)
Supplement: Supplementary file 2 — Supporting information S2. Data trace from the neckband system. [file JVIM-37-1475-s002.pdf]

Supporting information S2. Data trace from the neckband system.

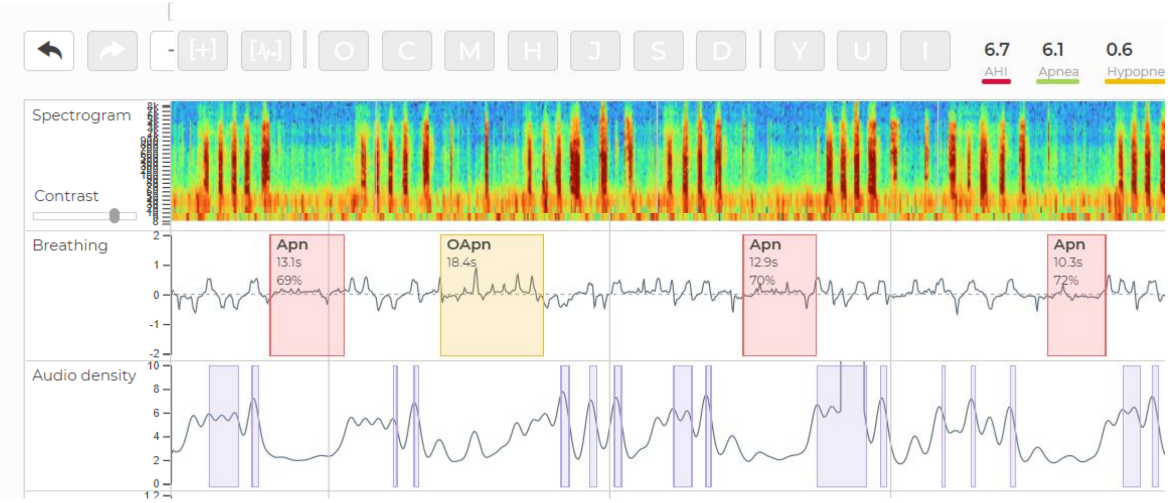

A three-minute epoch of respiratory tracing showing four obstructive apneas (automatically detected events marked as Apn., manually scored apneas OApn.).

First trace: Spectrogram of the sound signal.

Second trace: Breathing movements derived automatically from the tracheal sound signal.

Third trace: Audio density with marked snoring events.
